# State of the art in epitope mapping and opportunities in COVID-19

**DOI:** 10.2144/fsoa-2022-0048

**Published:** 2023-03-06

**Authors:** Samira M Hamed, Masarra M Sakr, Ghadir S El-Housseiny, Reham Wasfi, Khaled M Aboshanab

**Affiliations:** ^1^Department of Microbiology & Immunology, Faculty of Pharmacy, October University for Modern Sciences & Arts (MSA), Giza, 12451, Egypt; ^2^Department of Microbiology & Immunology, Faculty of Pharmacy, Ain Shams University, Cairo, 11566, Egypt

**Keywords:** diagnostic tools, epitope-based vaccines, epitope mapping, host-pathogen interface, SARS-CoV-2

## Abstract

The understanding of any disease calls for studying specific biological structures called epitopes. One important tool recently drawing attention and proving efficiency in both diagnosis and vaccine development is epitope mapping. Several techniques have been developed with the urge to provide precise epitope mapping for use in designing sensitive diagnostic tools and developing rpitope-based vaccines (EBVs) as well as therapeutics. In this review, we will discuss the state of the art in epitope mapping with a special emphasis on accomplishments and opportunities in combating COVID-19. These comprise SARS-CoV-2 variant analysis versus the currently available immune-based diagnostic tools and vaccines, immunological profile-based patient stratification, and finally, exploring novel epitope targets for potential prophylactic, therapeutic or diagnostic agents for COVID-19.

## Plain language summary

Epitope mapping is an important tool recently proving efficiency in both diagnosis and vaccine development. Several epitope mapping techniques have been developed for designing sensitive diagnostic tools and developing rpitope-based vaccines (EBVs) as well as therapeutics. In this review, we will discuss the state of the art in epitope mapping, emphasizing accomplishments and opportunities in combating COVID-19. These comprise SARS-CoV-2 variant analysis versus the currently available immune-based diagnostic tools and vaccines and exploring novel epitope targets for potential prophylactic, therapeutic or diagnostic agents for COVID-19.

Our immune system is classically categorized into arms: innate and adaptive immunity. All multicellular existences show some sort of innate immunity, which encompasses nonspecific defense mechanisms that play instantly after a microbe enters the body. Contrarily, specific adaptive immunity, which only exists in vertebrates, is expressed by B and T lymphocytes, which are responsible for humoral and cell-mediated immunity, respectively. Lymphocytes do not distinguish whole pathogens, but molecular components are identified as antigens [1]. Epitopes or antigenic determinants are the antigen parts that bind to antibodies or to membrane receptors on lymphocytes, thus causing either humoral or cellular immune response [2]. They are short amino acid sequences that can stimulate an immune response, more direct and powerful than that stimulated by the whole protein [3]. Epitopes signify the smallest wedge spotted by the immune system [4].

The discovery of epitopes, or epitope mapping, particularly for comparatively uncharacterized microbes, is a fundamental stage in the perception of viral pathogenesis and in discovering diagnostic reagents and epitope-based vaccines (EBVs). The idea of forming a vaccine from selected few epitopes has developed as a more rational tactic than conventional approaches since the latter are time consuming and antigen selection is quite arbitrary. In the previous 5 years, several novel vaccine candidates based on B-cell epitopes (BCE) and T-cell epitopes (TCE) have been suggested [1]. Since SARS-CoV-2, the causative agent of COVID-19, is a newly uncovered virus, research about antibody-eliciting epitopes and T-cell response is still ongoing [5–8].

Currently, literature is rich with review articles about the methods of epitope mapping and others about COVID-19 therapeutics, diagnostics and vaccines. However, in this review, our main aim is discussing epitope mapping as a tool for managing different aspects of COVID-19 pandemic. This is an attempt to link reviews discussing epitope mapping to the huge number of studies in which epitope mapping was applied in different aspects of COVID-19, hence covering an existing literature gap. We will first run through the different types of epitopes and overview the diverse methods of epitope mapping before highlighting the applications of epitope mapping in COVID-19 and setting an outlook for future perspectives. We believe this review may offer a starting point for researchers wishing to use epitope mapping in any of the covered applications.

## Types of epitopes

According to the sequential continuity, epitopes can be classified as linear (or continuous) and conformational (or discontinuous) epitopes. Linear epitopes are linear peptide segments comprised of a continuous stretch of residues along the polymer chain which are typically amphipathic helical 9–12 mers. Conformational epitopes are structurally more complex, nonlinear and distinct 15–22 mers residues with sequential discontinuity but spatial vicinity. They are composed of numerous successively discontinuous fragments aggregated together by the antigen folding into its native structure [2,9,10]. Linear epitopes are mostly on polysaccharides, fibrillar proteins and single-stranded nucleic acids. The antigen antibody reaction is dependent on the linear or primary structure of amino acids and they become available for antibody reaction upon denaturation of proteins in most cases. On the other hand, conformational epitopes are located on most globular proteins and native nucleic acids and antibodies recognize a conformational epitope by its specific three-dimensional structure [11].

Epitopes are divided into B- and T-cell epitopes, according to their corresponding receptors. Epitope recognition by B- and T-cells vary significantly. B-cells recognize antigens through membrane-bound immunoglobulins called B-cell receptors (BCR). The epitope binding site named paratope is sited at each tip of the two Fab fragments comprising the B cell receptor [12]. B-cell epitopes may be any exposed solvent region in the antigen and can be of diverse chemical nature, i.e., they can be small, whole chemical compounds or components of larger macromolecules like proteins, nucleotides, lipids and glycans [9]. Nevertheless, most antigens are proteins [1]. B cell epitopes can be categorized based on their immunogenic potency into: immunodominant (2–3-fold antibody level increase after vaccine administration), immunogenic (one-fold) and non-immunogenic (zero-fold) [2].

Conversely, T cells exhibit T-cell receptor (TCR) that recognizes T-cell epitopes when they are displayed attached to major histocompatibility complex (MHC) molecules on the antigen-presenting cells' (APCs) surface. T-cell epitopes are presented by MHC I and MHC II molecules that are identified by CD8^+^ and CD4^+^ T-cells, respectively. Accordingly, there are CD8^+^ and CD4^+^ T-cell epitopes [1]. T-cell epitopes are usually derived from protein antigens [11].

While most T-cell epitopes are linear, B cell epitopes include both linear (10%) and conformational (90%) epitopes. Earlier reports have shown that the majority of B-cell epitopes are conformational, which explains the complexity of B-cell epitope identification [2,10].

## Methods of epitope mapping

B- and T-cell epitopes can be determined by experimental methods. However, these methods are expensive and time consuming. Therefore, scientists have developed different epitope prediction methods utilizing the advances in immunoinformatic databases and tools. These epitope predictive methods have made epitope mapping easier and decreased the experimental effort and cost [1].

### Experimental determination of epitopes

#### T-cell epitope mapping methods

Potential T-cell epitopes could be detected by several direct and indirect methods. Direct methods detect binding of epitopes to TCRs using synthetic MHC molecules with bound epitopes. Such methods include; MHC multimers (MM), pepscan, and phage display peptide assay. On the other hand, indirect methods can detect indicators for T cells activation such as: cytokine production using solid phase MHC–peptide complexes, intracytoplasmic cytokine staining (ICS), enzyme-linked immunosorbent assay (ELISA) and enzyme-linked immunospot (ELISpot); activation-induced markers; activated cell multiplication using lymphoproliferation assay. Pepscan and phage display peptide assays can also be used for determination of B-cell epitopes.

##### Direct binding methods for T-cell epitope mapping

###### MHC multimers (MM)

Recombinant MHC molecules are synthesized in prokaryotic or eukaryotic cells. Tested peptide is considered potential epitope if the antigen-bound synthetic MHC molecule is recognized by TCRs [13]. The most commonly used multimers are composed of four streptavidin MHC molecules (MHC tetramers) covalently attached to biotin [14]. This simple method offers an advantage for collecting T cells for reuse in flow cytometry studies. However, it requires previous knowledge of MHC–peptide binding as well as possible epitopes [15].

###### Pepscan

The pepscan method is a peptide microarray-based technique and it is used to identify epitopes. In this technique, a protein or protein fragment is sliced into a series of overlapping fragments of fixed size. These overlapping fragments are then fixed to a support to test their binding to antibodies or T cells. This binding is measured using different label-free methods such as surface plasmon resonance and mass spectrometry [16], or label-dependent ELISA assay followed by data analysis by bioinformatics. The majority of peptide arrays are analyzed using label-dependent methods [17].

###### Combinatorial phage display method

This method includes screening of phage-displayed random peptide libraries for their binding to immobilized antibodies or T-cells. After extensive washing, phages displaying the bound peptide are released for further amplification [18].

##### Indirect methods for T-cell epitope mapping

###### Detection of cytokines produced by activated cells

####### Solid-phase MHC–peptide complexes

In this method, MHC class I molecules are spotted on a microarray slide and allowed to bind to possible peptides that can be recognized by the T-lymphocytes. Fluorescent-labelled anti-cytokine antibody is then added to the microarray for detecting activated T-lymphocytes [19].

####### Intracytoplasmic cytokine staining

This method is a flow cytometry-based method. Different cytokines produced by activated T-lymphocytes are detected by monoclonal antibodies labelled with different colors specific to different cytokines, as described by Letsch A, Scheibenbogen C [20]. This method is expensive and requires professional experience [21].

####### Enzyme-linked immunosorbent

This method is a sandwich ELISA technique in which wells are coated with anti-cytokine antibody, then tested antigen followed by stimulated T lymphocytes. After Incubation, activated T cells will release specific cytokine which bind to well-bound antibodies and then detected by secondary labelled antibody. This technique can be used for both qualitative and quantitative detection of cytokines [22].

####### Enzyme-Linked Immunospot (ELISpot)

This is a sandwich ELISA technique which is a modified form of the cell ELISA assay used for detection of CD4^+^ and CD8^+^ cell activation [23]. The modification includes using enzyme substrate which is converted to insoluble compound by the enzyme linked to secondary antibodies. Insoluble colored products appear as a spot reflecting the number of cytokine-producing cells. ELISpot technique is used to quantify individual cytokine-secreting cells; therefore it is more sensitive than ELISA assay. The ELISpot technique is widely used because it requires simple devices to detect end point, however it cannot be used solely for detection of cytokine and is usually used in addition to ELISA technique [22].

###### Activation-induced markers

In contrast to the previously described methods which can detect activation of T helper cells only, the activation-induced marker method can detect activation of CD8^+^ cells as well as CD4^+^ cells. In this method, the peripheral blood mononucleocyte stimulated with an antigen is incubated, then CD markers developing upon T-cell activation are stained by specific antibody and measured using flow cytometry [24].

###### Detection of activated T-cells multiplication by Lymphoproliferation assay

In this assay, activated T lymphocytes are detected by their increased proliferation rate. The increase in the number of activated T-cells is reflected by an increase in DNA production and consequently an increase in radiolabeled thymidine incorporation by the cells [25]. Despite some inherent drawbacks, this method is widely used in epitope mapping owing to its high sensitivity [17].

#### B-cell epitope mapping methods

B-cell epitope mapping is complementary to T-cell epitope mapping in developing vaccines for viral infections [21,26]. Results of antibody-bound epitopes should be carefully considered due to several reasons: the results of antigen neutralization *in vivo* could be different from *in vitro* results [27]; antibody binding could increase the infectivity of host cells to viruses *in vivo* by antibody-dependent enhancement (ADE) [27]; and finally, many epitope mapping methods cannot differentiate between discontinuous and linear epitopes [28]. B-cell epitope mapping methods may utilize antigen fragments, recombinant and synthetic antigens or modified antigens.

##### Methods utilizing native antigen or antigen fragments

###### Crystallography-based methods

This method is one of the early techniques in epitope mapping. In this technique, the highly purified antigens are allowed to co-crystallize with their corresponding antibodies. This complex is then visualized and analyzed using x-ray diffraction [29,30].

###### Cryo-electron microscope-based methods

The cryo-electron microscopy is a Nobel-prize-winning technique, which is used for studying virus-antibody complexes with high resolution. This method overcomes the limitations of the x-ray diffraction method because crystallization of the virus-antibody complex could be challenging in some cases. In this technique, immune complexes are frozen in a thin layer of vitreous ice to maintain their original conformations which improve their analysis at a high resolution. The complexes are then analyzed using direct electron detection camera (DED) [31].

###### Mass spectrometry-based methods

This method has improved the identification of discontinuous epitopes [32]. Antigens are subjected to lysis by different proteases. The released fragments are then detected by mass spectrometry (MS) in presence and absence of antibodies [33].

###### Nuclear magnetic resonance (NMR)

This method can be used for explaining the nature of epitope recognition by giving more details on epitope-antibody binding using NMR analysis. This tool can be used for the atomic-level characterization of the intermolecular interfaces between the antigen and the antibody. Epitope residues are determined by studying the difference in the NMR signal of the free antigen compared with that bound to an antibody [29].

###### Binding assays

Binding assays can be used to study the qualitative and quantitative binding of antibodies to antigen fragments whether protein or polysaccharide [29]. The binding can be studied using western blot [34], dot plot [29], ELISA techniques [35] or a combined form of the mentioned techniques [36]. This method can be applied to screen individual epitopes in a mixture [28].

##### Methods utilizing recombinant & synthetic antigens

###### Peptide microarray (PMA)

In the PMA method, synthetic peptide is prepared then spotted on nitrocellulose coated glass slide for fixation. Antibodies that bind to the fixed peptides are detected using a secondary labelled antibody. This method offers strong peptide binding capacity and long-term stability for bound peptides [10].

###### Surface plasmon resonance

SPR is a highly sensitive technique that can be used to study the real-time interaction of biological molecules in one step without labelling of antibodies [37]. Binding of the antibody to the epitope is reflected by a shift in the reflectivity band of the bound antibody compared with the unbound one. Three methods could be used for biomolecule binding to the surface of sensor chips. These include covalent immobilization, affinity capture, and hydrophobic adsorption. Of them, the affinity capture is the most suitable method for studying monoclonal antibodies [38].

##### Methods utilizing modified antigens

This method is based on making a site-directed mutagenesis in the antigen which can be useful for identifying the amino acid residues involved in the antigen binding to the antibody. The change in antibody binding after certain mutations in the antigen can be used to determine the genuine epitope of this antigen [39]. This is a popular method for epitope mapping due to its relative simplicity [28].

### *In silico* epitope prediction

#### T-cell epitopes

Recognition of epitopes by T cells occurs by the TCR. These epitopes must be displayed on the surface of APCs bound to MHC molecules. T-cell epitopes are presented by MHC I and MHC II molecules that can stimulate CD8^+^ and CD4^+^ T-cells, respectively. The size of epitopes presented by MHC molecules is restricted depending on the processing pathway and the MHC binding. Therefore, prediction of peptide-MHC binding is the main basis to anticipate T-cell epitopes and the high degree of MHC polymorphism makes T-cell epitope prediction challenging [40,41]. The *in silico*-based prediction methods decrease the number of antigenic peptides for experimental testing, thus saving time and cost [42]. They usually offer accurate results for T-cell epitopes because the majority of T-cell epitopes are linear [43], in contrast to the discontinuous B-cell epitopes which could give unreliable results in some cases. The difference in peptide binding grooves of MHC I and MHC II leads to a difference in antigen binding to these molecules. The binding groove of MHC II is open at both ends. Therefore, the length of the peptides that bind to class II molecules can range from 13 to 25 amino acids compared with 8–10 amino acids for class I molecules. Consequently, peptide-binding prediction of the correct 9 mer core residues in the long peptide bound to MHC II molecules is less accurate than that of MHC I molecules [44,45].

*In silico*-based methods for prediction of peptide-MHC binding can be divided into data-driven and structure-based methods. The data-driven methods are based on peptide sequences that are known to bind to MHC molecules and are listed in the specialized epitope databases such as IEDB, EPIMHC, and Antijen (Table 1 [45]). The MHC polymorphism among different individuals is one of the obstacles to T cell prediction by MHC binding models, therefore advances in artificial intelligence has been utilized in Peptide-MHC binding prediction methods [46]. Machine learning models were superior to other *in silico* prediction methods [46,47].

**Table 1.** *In silico* prediction tools of T and B-cell epitopes.

Epitope type	Method of measurement	Description of method	Prediction tool	URL	Ref.
T-cell epitope mapping	Data driven	Prediction of peptide-MHC binding based on sequence motif (SM) that depends on detection of MHC-binding amino acid motifs which are called anchor residues	MotifScan	https://myhits.sib.swiss/cgi-bin/motif_scan	[46]
		Evaluate the contribution of all peptide positions to MHC molecule binding	Rankpep	http://imed.med.ucm.es/Tools/rankpep.html	[199]
			SYFPEITHI	http://www.syfpeithi.de/	
			PEPVAC	http://imed.med.ucm.es/PEPVAC/	
			EPISOPT	http://bio.med.ucm.es/episopt.html	
			Vaxign	https://violinet.org/vaxign2	
		This approach is quantitative affinity matrices (QAMs) which involves peptide binding affinity scores	BIMAS2	https://edumetrisis.com/bimas-2/	[200,201]
			Propred	https://mybiosoftware.com/propred1-propred-predict-mhc-class-i-class-ii-binding-regions-antigen-sequence.html	
			EpiJen	http://www.ddg-pharmfac.net/epijen/EpiJen/EpiJen.htm	
	Structure based method	In the QSAR model, the binding affinity of peptides to MHC is computed as the sum of amino acid contributions at each position plus the contribution of adjacent peptide to anchor site	MHCPred	http://www.ddg-pharmfac.net/mhcpred/MHCPred/	[202,203]
			EpiDOCK,	http://www.ddg-pharmfac.net/epidock/	
	Machine learning models	Pan-MHC specific methods made by training artifical neural network (ANNs) on input data including MHC residues that contact the peptide and peptide-binding affinity which can be used in predicting peptide-binding affinities to uncharacterized human leukocyte antigen (HLA) alleles	NetCTL	https://services.healthtech.dtu.dk/service.php?NetCTL-1.2	[204]
B-cell epitope mapping	Machine learning models	3D structure-based methods	DiscoTOP	http://tools.iedb.org/discotope/	
			ElliPRO	http://tools.iedb.org/ellipro/	
			SEPPA	http://www.badd-cao.net/seppa3/index.html	
			EPITOPIA	http://epitopia.tau.ac.il/	
			CBTOPE	http://crdd.osdd.net/raghava/cbtope/	
			EPCES	http://sysbio.unl.edu/EPCES/	
			PEASE	https://maayanlab.cloud/datasets2tools/landing/tool/PEASE	

#### B-cell epitopes

Earlier prediction methods for B-cell epitopes were unsuccessful in practical use. Therefore, advances in machine learning has developed the *in silico* methods of prediction [48]. *In silico* prediction methods are classified according to the input data into: antigen sequence- and 3D structure-based methods. It was found that the 3D structure-based methods of prediction are superior to the sequence-based methods (Table 1) [49].

## Applications of epitope mapping in COVID-19

**Figure 1.**
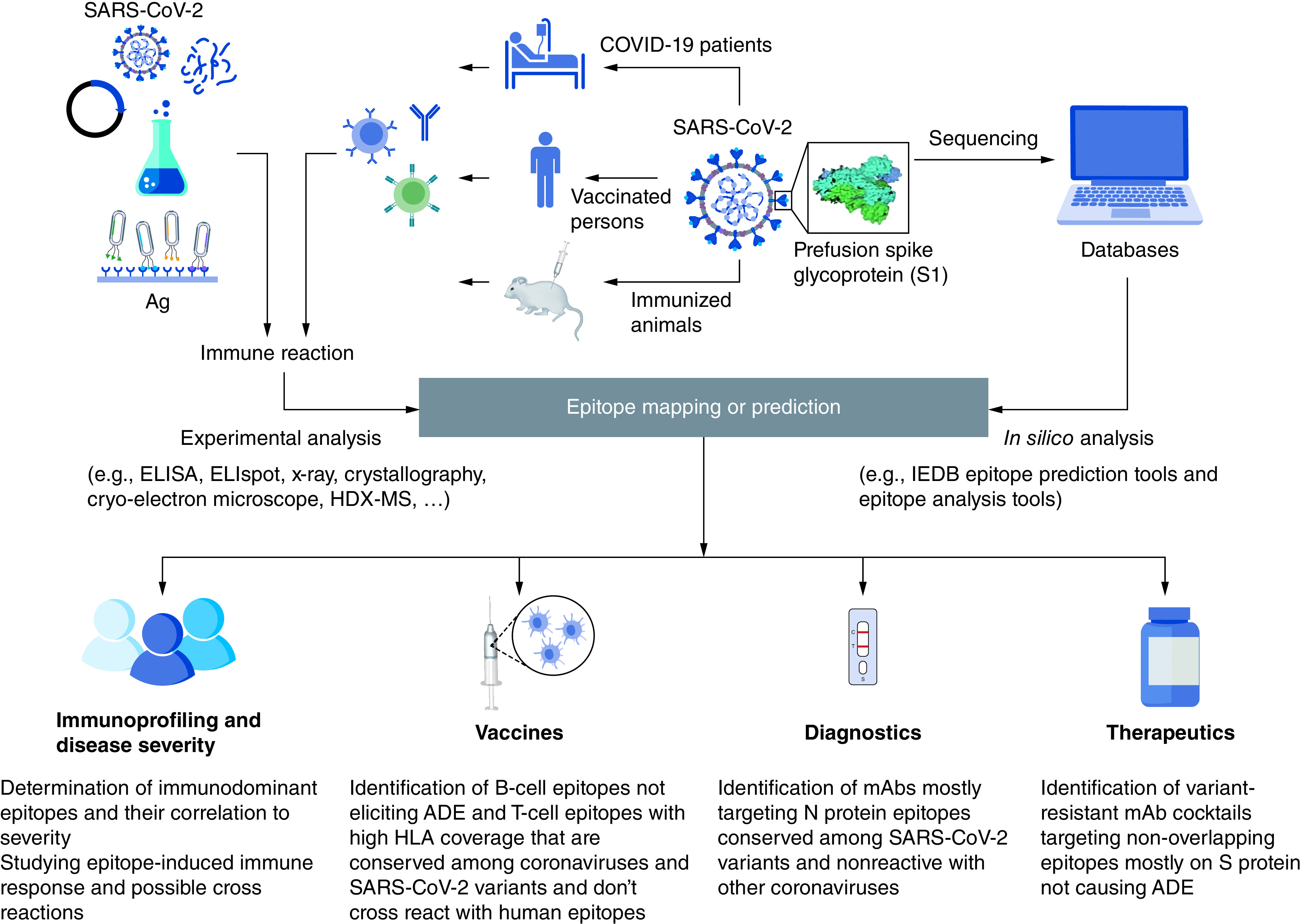
Representation for epitope mapping techniques and its significance in understanding disease severity, preparation of diagnostics, vaccines as well as therapeutics. Both *in silico* epitope prediction tools and experimental epitope mapping are used for identification of SARS-CoV-2 epitopes. Epitope prediction and analysis tools are fed by protein sequences available in protein databases for prediction of potential epitopes that are then filtered for selected features. For experimental epitope mapping, SARS-CoV-2-specific antibodies, B-cells or T-cells may be isolated from COVID-19 patients, vaccinated persons or immunized animals. The interaction with whole, fragmented, synthetic, or recombinant antigens as well as phage display libraries can be analyzed using several experimental methods such as: ELISA, ELISpot, Cryo-electron microscope, etc. ADE: Antibody-dependent enhancement.

Identification of epitopes have proven to be a pivotal step in the understanding of disease progression, and for the development of EBVs, diagnostic tools, and therapeutic antibodies. Figure 1 shows a schematic representation displaying how epitope mapping and prediction could be done in addition to its various applications. Since the beginning of the COVID-19 pandemic, a lot of research has been conducted to fill the knowledge gap and provide opportunities for controlling the worldwide spread of SARS-CoV-2.

### Epitope mining & variant analysis

#### SARS-CoV-2 structure

**Figure 2.**
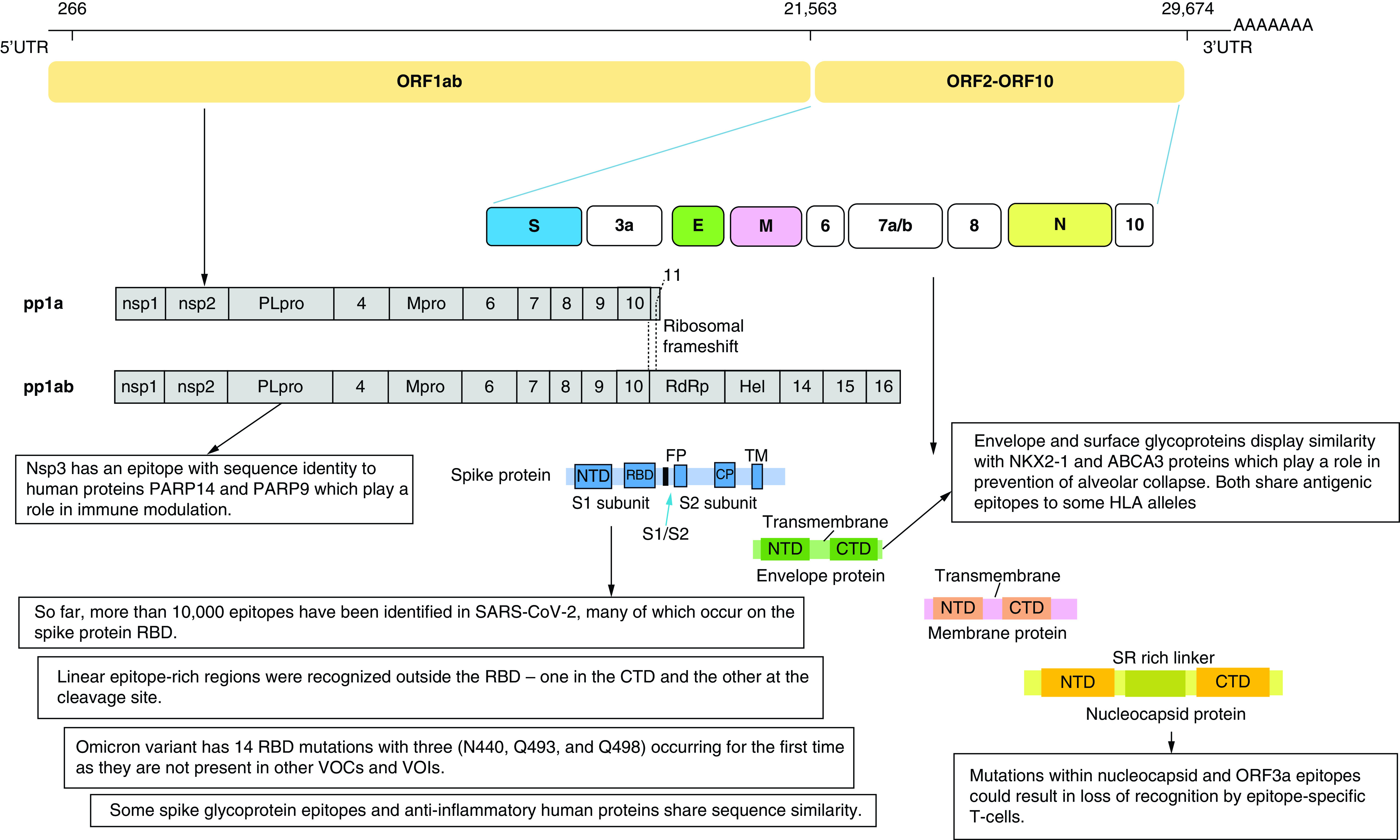
SARS-CoV-2 genome showing the produced structural and non-structural protein with focus on some major findings of epitope mapping. Epitope mining has revealed the presence of several immunodominant epitopes on the RBD as well as other regions of the Spike protein. Mutations in these epitopes have resulted in new variants of SARS-COV-2. Mimicry of some viral epitopes with other human proteins could provide an explanation for hyperinflammatory response. CTD: C-terminal domain; NTD: N-terminal domain; RBD: Receptor binding domain.

At the beginning of the pandemic, the limited information about SARS-CoV-2 epitopes and the protective immune response against it represented an obstacle hindering vaccine development, therapeutics, as well as diagnostic agents. Several studies rushed to mine for epitopes on SARS-CoV-2 and to gather serological information from patients. SARS-CoV-2 is an enveloped, single-stranded RNA virus. Its genome encodes 27 proteins, 4 of them are the main structural proteins: the Nucleocapsid (N), Spike (S), Envelope (E) and Membrane (M) proteins. The S glycoprotein is a transmembrane protein and is split into 2 subunits, S1 and S2, by a host cell protease. Figure 2 shows the genome organization and the structure of SARS-CoV-2. The S1 subunit contains the C-terminal domain (CTD) holding the receptor binding domain (RBD), making it the target most focused on when studying the epitope displays and virus-host interactions. Sixteen non-structural proteins (nsps) are also encoded by ORF1a and ORF1b containing replication and transcription related genes [50].

#### Determination of immunodominant epitopes & comparison to SARS-CoV

Early on, a study analyzed the correlation between Spike (S) and Nucleocapsid (N) protein-specific antibody levels and identified immunodominant sites. Nine major linear B- cell immunodominant (ID) sites on SARS-CoV-2 Spike protein were determined, four of which on the receptor binding domain (RBD) [8]. Several other studies identified immunodominant linear epitopes on the S protein [51–53]. One study reported a total of 62 T-cell epitopes were predicted to be localized on the S protein of SARS-CoV-2, 40 and 22 peptides as potential CD8^+^ T-cell epitopes and CD4^+^ T-cell epitopes, respectively. Three of these epitopes have been reported as T-cell epitopes in SARS-CoV S protein [53].

Immunoinformatic analysis of SARS-CoV-2 B-cell epitopes displayed the presence of 9 linear B-cell epitopes on the S protein of SARS-CoV-2 as opposed to 7 previously reported linear B-cell epitopes on the S protein of SARS-CoV. Even though the primary structure of the S proteins from both viruses are highly similar, the two groups of epitopes displayed almost no similarities. All the nine epitopes were localized to a conserved region on the surface of either the monomer or trimer of the S protein [53]. A more recent study however, reported that antibodies against SARS-CoV-2 can cross react with other human coronaviruses epitopes [7].

Although several studies reported the presence of linear immunodominant sites in RBD, recently a landscape of SARS-CoV-2 linear epitopes constructed from more than one thousand patients reported that RBD lacks linear epitopes. Knowing that RBD of the SARS-CoV-2 Spike protein is highly immunogenic, the study assumes that epitopes of the RBD are mostly conformational [54]. In accordance with this, another study reported the lack of linear epitopes toward Spike RBD and an abundance of structural mimotopes [55]. An early study had primarily identified a peptide, (S1–82), located on the surface of receptor-binding-motif (RBM) as an epitope; however, it was afterward revealed to lack high specificity. No significant binding was reported for the rest of the RBD located peptides, suggesting that conformational epitopes are dominant for this region [56].

Epitopes outside the RBD region were also identified [51,53,54]. Two linear epitope rich regions were recognized outside RBD- one in the CTD and the other at the cleavage site [54]. The presence of epitopes at key proteolytic sites suggests they are critical for cell entry. One of the epitope rich regions overlaps with the fusion peptide site and the proteolytic cleavage site (S20) suggesting that targeting this epitope-rich site could lead to ultimate blockage of membrane fusion during viral entry (Figure 2) [53,57].

In addition to linear epitopes, multiple discontinuous conformational B-cell epitopes that were detected on eight regions located on the trimeric S protein surface, ten of which are potentially involved in the binding of RBD to human angiotensin converting enzyme (ACE2) [53].

The immune epitope database (IEDB) contains a list of all validated B and T-cell epitopes. As of 5 May 2022, 10,438 experimentally tested SARS-CoV-2 epitopes were deposited in the IEDB., many of which occur on the Spike protein [58]. These included 8,202 B-cell epitopes (7,530 linear and 672 discontinuous) and 2,526 T-cell epitopes most of them (2504) are linear epitopes. T-cell epitopes were most frequently mapped using biological activity, ICS, MM, and ELIspot assays. For B-cell epitope mapping, microarray, ELISA, neutralization assays, and SPR were most commonly used.

#### SARS-CoV-2 variant analysis

With the presence of an unprecedented number of SARS-CoV-2 sequences available, several studies for the analysis and characterization of the SARS-CoV-2 genome variants were conducted. Variant analysis is important for identifying the widely spread clades, investigating mutation sites and for effective vaccine development.

A Study that analyzed over 229,000 SARS-CoV-2 sequences identified over 39,000 variants worldwide, many of which were non-sense variants [59]. Assessing the Spike protein variants 427 non-synonymous variants, the majority of which were found to be located in the RBD and B-cell epitopes [60]. The clade referred to as D614G clade was reported to be the most common [55,60,61]. In this strain, a glycine replaced aspartic acid at the position 614 of Spike protein of the initially prevailing 614D strain. This was associated with higher viral loads in infected young patients with no increase in the severity or mortality [61]. A study that analyzed 12,343 SARS-CoV-2 genome sequences isolated from patients however, reported significant positive correlations between ORF1ab 4715L and S protein 614G variants and fatality rates [62]. Most of the amino acid changes across SARS-CoV-2 variants were found in the B-cell epitopes of the RBD and N-terminal domain (NTD) of the S1 subunit as well as S1/S2 cleavage site [63].

### Immuno-profiling & disease severity

#### Epitope-antibody response

Epitope responses are known to affect disease severity in many cases. Moreover, studying epitope-antibody interaction is known to be crucial in understanding disease development. Identification of neutralizing antibodies (nAbs) has hence become a focal point in SARS-CoV-2 research. Studies reported SARS-CoV-2 epitopes that elicited both neutralizing and non-nAbs [54,56,57,64].

While the role of nAbs seems understandable, non-nAbs are also essential in combating the infection. Any virus-binding antibody is considered beneficial and helps in eliminating the pathogen through antibody-dependent enhancement and antibody-dependent cellular cytotoxicity [54]. A study that used an animal model to investigate the role of non-nAbs revealed that maximum opsonization level could be achieved using plasma and patient-derived monoclonal antibodies from convalescent patients at low levels of bound antibodies. This was reduced as antibody binding to Spike protein increases. Non-nAbs are believed to provide protection against SARS-C-V-2 through mediating phagocytosis.

A recent study that used *in vivo* model mentioned that in addition to neutralization, additional antibody properties like Fc-mediated effector functions from a non-nAb can confer protection against SARS-CoV-2 infection and spread [65]. In the same study, epitope analysis identified several antibodies that bind to RBD or NTD (denoted Ab11, 57, 59, 66, 77, 81). The antibody Ab59 was found to be a nAb whereas Ab94 that could bind with both RBD an NTD, along with other antibodies were non-neutralizing. Neutralizing antibodies inhibit Spike-ACE2 interaction; however, too high doses of nAbs were found to be not optimum in the animal treatment model [64]. A study also reported the presence of 11 nAbs with good binding affinities to epitopes in the RBD of the S protein and suggested they could be tested as therapeutic candidates [52]. Several reports mentioned that RBD-binding antibodies constitute the majority of the neutralizing activity observed in plasma of convalescent patients [66,67].

Immuno-profiling identified that peptides S456–460 as well as S455–469 contain an identical linear B-cell epitope. These peptides overlap with ACE2-binding residues [68,69]. The antibody elicited by S455–469 was found to have a neutralizing effect on SARS-CoV-2 pseudovirus indicating it is a probable neutralizing epitope [69,70]. Furthermore, immunization of mice with RBD-based antigens where linear B-cell epitope peptides were used showed the immunogenicity of three immunodominant peptides displayed by their ability to elicit potent antibody response to SARS-CoV-2. Neutralization assays revealed that the corresponding antibodies had neutralization activities. One of these tested peptides, R450 (S450–469), contained the same linear B-cell epitope that occurs in the peptides S456–460 and S455–469, indicating it is a potential neutralizing epitope [71].

Structural analysis of the SARS-CoV-2 S trimer revealed that ^350^VYAWN^354^ was a highly conserved epitope on the surface of the S trimer, whereas ^473^YQAGSTP^479^ located in the RBM displayed variability among different SARS-CoV-2 strains and was hugely different to SARS-CoV and MERS-CoV. The linear B-cell epitope ^473^YQAGSTP^479^ and both of 473Tyr and 479Pro are believed to be important residues for epitope-antibody interaction [71].

Characterizing the epitope landscape of SARS-CoV-2 antibodies using a peptide-based proteome microarray reported the presence of two antibodies which bind to linear epitopes on RBD. One of them, a rabbit monoclonal antibody, displayed significant inhibitory activity. It was found to be able to bind to five epitopes on the RBD domain [72]. However, one of the studies that contradicted the presence of linear epitopes on RBD of S protein mentioned that antibodies from mice immunized with the linear peptides exhibited no significant neutralization activity concluding that little neutralization activity was reported for linear-epitope- elicited antibodies [54]. It is therefore clear that further investigations are still required to confirm the presence of linear epitopes on RBD region and determine their roles.

Limiting the epitope-antibody analysis to only the RBD region is very risky as potential mutations and viral evolution might happen. Accordingly, identification of epitopes and domains outside RBD region that can elicit nAbs has also been an important aspect of research. Of these, the peptides derived from the FP region (S2–18, S2–19 and S2–20) showed ability to elicit nAbs activity [54]. This fusion peptide is a short segment of about 15–20 amino acids which was shown to play a role in membrane fusion during viral cell entry [73].

Investigating the activity of isolated nAbs from patients severely infected with SARS-CoV-2 showed that nineteen out of sixty-one antibodies potently neutralized SARS-CoV-2 *in vitro*. Epitope mapping showed that about half of these tested antibodies were directed against RBD and the other half against the N-terminal domain (NTD), indicating that both regions of the Spike protein are highly immunogenic [74].

#### Epitope-antibody response & disease severity

Studies showed that higher antibody titers were also found to be associated with more severe conditions [75]. A positive correlation existed between responsive epitope numbers and disease severity. Significantly higher numbers were found in the severe groups as compared with the non-severe group. Some epitopes were found to correlate with severity of the disease. A study correlating the epitope-antibody interactions to the severity and fatality of the disease reported a significant reduction in the IgG response for some epitopes on the S protein – namely S1–93, S1–9, and S2–78 – in the non-survivor group, suggesting protective roles for the corresponding antibodies [54].

Immune response to viral epitopes is well known to either take place by direct B-cell recognition or by T-cell mediated immunity where viral antigens are presented by major histocompatibility complexes (MHC) to the T-cells followed by antibody production and cytokine secretion. The well-known polymorphism in MHC was found to be correlated with the disease severity in many ways [76]. More than 120 immunogenic and immunodominant SARS-CoV-2 T-cell epitopes have been reported [77]. Several hundred different HLA class I and II restricted SARS-CoV-2-derived epitopes have been identified [78]. It was also observed that patients with severe diseases showed significantly larger SARS-CoV-2-specific T-cell populations as compared with patients with mild diseases. This suggests that antigen-specific T-cell responses are associated with different disease outcomes [77]. An inverse association was observed between total predicted MHC-I epitope and COVID-19 mortality [79].

Epitope mimicry analysis revealed that respiratory failure in population with some MHC polymorphisms was due to the high similarity between envelope and surface glycoproteins and two human proteins important for the prevention of alveolar collapse and respiratory failure, NKX2-1 and ABCA3 proteins. Both share antigenic epitopes to some HLA alleles (Figure 2) [80]. However, this finding needs yet to be experimentally validated.

**Figure 3.**
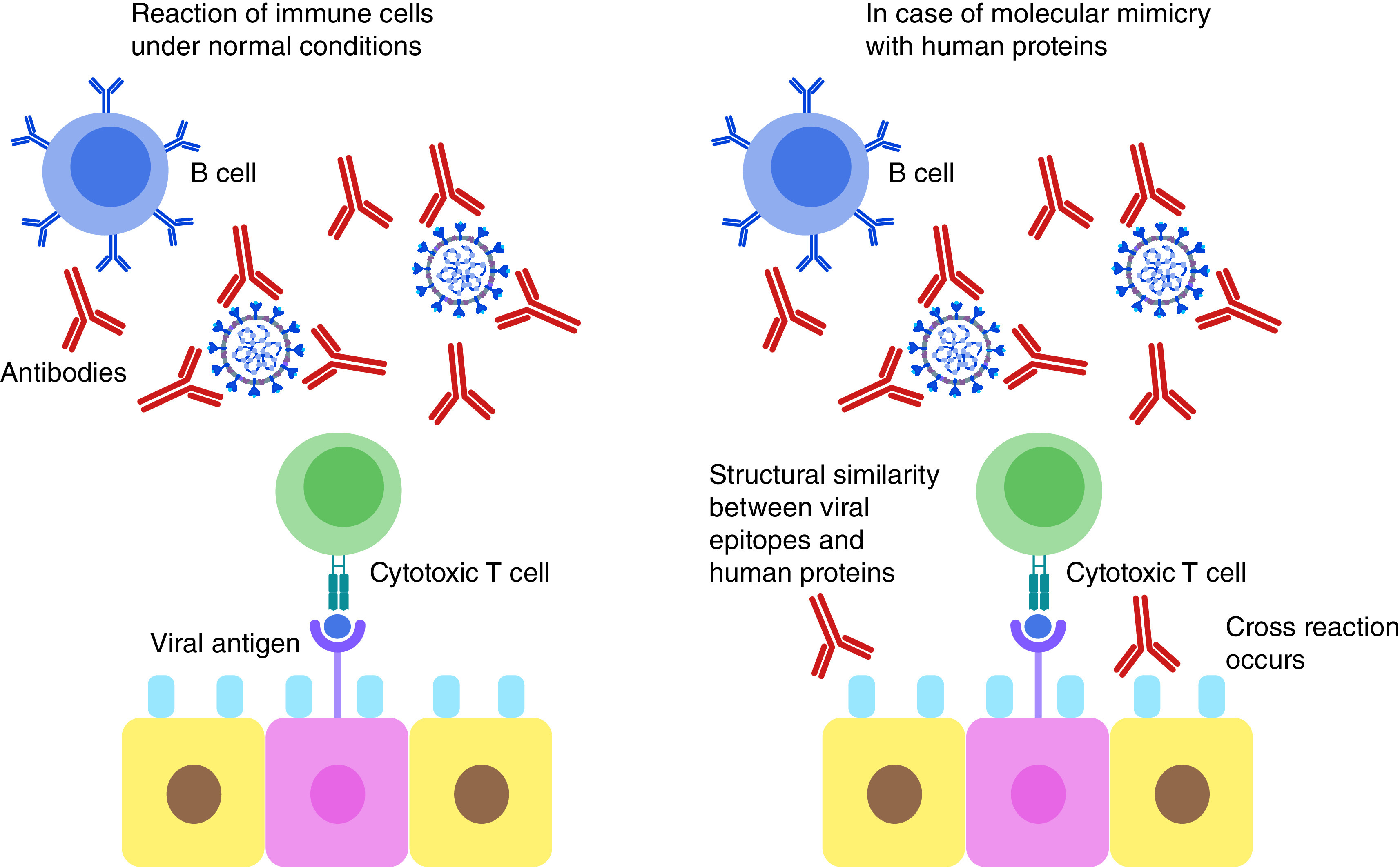
Mechanism of proposed molecular mimicry of SARS-CoV-2 epitopes and human proteins. This can lead to cross reaction with the immune cells resulting in hyperinflammatory response, cytokine storm and respiratory failure.

SARS-CoV-2 has been reported to often induce a cytokine storm that is characterized by diverse disorders such as systemic multiorgan hyperinflammation, endothelial dysfunction, macrophage activation syndrome, and acute respiratory syndrome. Explanation to this raging cytokine storm might lie in the possible cross-reactions between the SARS-CoV-2 Spike glycoprotein and anti-inflammatory human proteins (Figure 3). According to an *in silico* study by Kanduc D., analyzing the immunological potential of the peptide sharing revealed that almost all the shared pentapeptides occurring in SARS-CoV-2 Spike epitopes, except for nine, were immunoreactive validating the possibility of the previously mentioned cross-reactions [81]. It is also worth mentioning that many of these shared peptides are also present in other pathogens to which the individual might have been exposed to either by previous infection or vaccination and of which the immune system has stored memory. This might also contribute to a powerful cross-reactive response. Determining these shared pentapeptides in immunoreactive epitopes are clearly crucial for safe vaccine development [81].

A study implementing serum epitope repertoire analysis reported, on the contrary, that prevalent cross-reactive S protein epitopes were not correlated to COVID-19 severity but rather nucleoprotein epitopes This study mentioned that a shift toward Spike-directed antibody response in recovering patients was observed as compared with a nucleoprotein-directed antibody response in deceased patients. The study attributed the boosting immune response reported in many COVID-19 cases to the prevalent cross-reactive epitopes in the S2 subunit of the Spike protein of SARS-CoV-2 and other coronaviruses. According to this study, pre-existing antibodies resulting from previous exposure to human coronaviruses are responsible for this phenomenon [55]. A previous finding that supported this assumption was that cross-reactive epitope at Spike protein near the FP region could elicit antibody response in individuals uninfected with SARS-CoV-2 [82].

Another explanation for triggering a cytokine storm was attributed to an epitope of SARS-CoV-2 non-structural protein nsp3. This nsp3 epitope contains sequence identical to the human poly-adenosine diphosphate-ribose polymerase proteins (PARP14 and PARP9). These proteins play a role in immune modulation and are responsible for macrophage activation. This molecular mimicry may be responsible for the hyper-inflammatory condition but experimental validation is still required (Figure 2) [83]. N protein could also play a role in cytokine storm through inducing proinflammatory responses as it was found to promote IL-1β and IL-6 activation [84].

A computational model, as well, provided another possible explanation; a sequence motif unique to SARS-CoV-2 on one of the binding epitopes of the S protein was found to be highly similar in both sequence and structure to the bacterial superantigen enterotoxin B of *Staphylococcus sp*. suggesting that SARS-CoV-2 Spike protein can bind with high affinity to T-cell receptors and form a complex with major histocompatibility class II (MHC II) triggering a cytokine storm [85].

### Mutability monitoring & prediction

#### Detection of mutations in VOCs & VOIs

All viruses change over time. Although most changes have little effect on the virus properties, some cause dramatic changes. Monitoring and predicting epitope mutations are crucially important for guiding development of vaccines, diagnostics, and therapeutics. The emergence of new variants that can escape immune response and evade antibodies elicited by previous infections or vaccination will always pose a threat to public health [86]. Different tools have therefore been developed to track and predict mutations and determine their potential impact on the transmissibility and pathogenicity. Variants with epitope mutations affecting transmission, immune evasion, diagnostics, or therapeutics are classified as either variants of interest (VOI) or concern (VOC).

The WHO described the VOC as a SARS-CoV-2 variant that is associated with one or more of the following changes: an increase in transmissibility or COVID-19 epidemiology, an increase in virulence or change in clinical presentation of the disease or decrease in the effectiveness of public health measures including available diagnostics, therapeutics or vaccines while the VOI was described as a SARS-CoV-2 variant with genetic changes that are predicted or known to affect virus properties and at the same time identified to cause significant community transmission or multiple COVID-19 clusters [87]. The currently circulating VOCs include the Delta and Omicron variants while previous ones included Alpha, Beta and Gamma variants. There are currently no circulating variants of interest [87]. Among the recently tracked variants are Omicron variant, BA.5, XBB and BQ.1. However, WHO's Technical Advisory Group reported that they do not differ greatly from other Omicron lineages with additional immune escape mutations [88].

Until May 2021, only seven positions within the RBD domain were mutated in the VOI and VOC strains compared with the Wuhan-Hu-1 reference strain [88]. Later on, Omicron variant displayed 14 RBD mutations with three (N440, Q493, and Q498) occurring for the first time as they are not present in other VOCs or VOIs [89].

Identification of conserved epitopes among the VOCs and VOIs is therefore clearly important for effective vaccine development. An example to this is the highly conserved epitope identified in the S protein by Wang *et al.* which was reported to be in the circulating variants as well as other sarbecoviruses [90].

#### SARS-CoV-2 mutations & immune escape

Identification of mutations that escape binding by polyclonal antibodies has become a focal point providing provisional surveillance for SARS-CoV-2 evolution. One of the methods used yeast libraries expressing RBD mutants and incubated them with polyclonal human plasma to detect their binding affinities relative to the un-mutagenized RBD. This revealed that binding was affected by mutations in three main epitopes in the RBD. Single mutations in RBD that were mapped to strongly decrease binding of plasma antibodies were also found to greatly reduce viral neutralization. It also showed that E484 – despite heterogeneity within and between individuals – is the most important site for viral mutations where more than ten-fold reduction in neutralization was observed with several mutations including one in the emerging 20H/501Y.V2 and 20J/501Y.V3 SARS-CoV-2 lineages. Mutations within other epitopes (like the 443–450 loop, site 417, and residues around 484) were also found to affect antibody binding [91].

Studies also showed that mutations within nucleocapsid and ORF3a epitopes could result in loss of recognition by epitope-specific T cells. Polymorphism in HLA genes restrict the selective advantage of escape within one particular epitope to a relatively small proportion of the population. It is therefore unlikely for the polyclonal T-cell response to be significantly reduced in a given individual by mutations present in any one circulating variant. This is unlike the significant impact observed with nAb responses seen with epitope mutations [92].

Antibody-escaping mutations are already present in circulating variants. The Omicron variant has over 30 mutations in the S glycoprotein. Five of the mutations that occur in the RBD (G339D, N440K, S477N, T478K and N501Y) were previously reported to enhance binding to human ACE2, consequently, affecting viral transmissibility [93,94]. At least seven of the RBD mutations (K417N, G446S, E484A, Q493R, G496S, Q498R and N501Y) were found to influence antibody neutralization [92]. The NTD of the Omicron variant was also found to have four amino acid substitutions (A67V, T95I, G142D and L212I), three deletions (69–70, 143–145 and 211) and an insertion (EPE between 214 and 215) compared with the Wuhan-Hu-1 strain. These mutations involve a number of B and T-cell epitopes and are believed to reduce sensitivity of Omicron to neutralization by anti-SARS-CoV-2 antibodies induced by either infection or vaccination [93]. A recent study reported that reduced recognition to Omicron Spike was primarily observed within the CD8^+^ T-cell due to escape from HLA binding [94]. A study that assessed the ability of T-cell to recognize Omicron S protein, however, showed that between 70% and 80% of the CD4^+^ and CD8^+^ T-cell response to S protein was maintained in vaccinated individuals or convalescent patients. This indicates the ability of T-cell responses to recognize the Omicron variant despite extensive mutations and reduced susceptibility to nAbs [95]. Also broadly neutralizing monoclonal antibodies that can recognize conserved RBD epitopes were shown to overcome Omicron antigenic shift [96].

A recent study used ELISA to identify key epitopes within structural and non-structural proteins that can elicit a strong immune response. It was observed that patients with severe disease displayed a weaker IgG response to NS2 epitope identified in ORF8 than patients with mild or moderate conditions. This emphasizes that ORF8 is a unique and specific protein in SARS-CoV-2. A peptide obtained from M protein (M1) was also identified as a new epitope related to severity [97]. Reports also showed that non-synonymous mutations in MHC-I epitopes resulted in the formation of less stabilized MHC-I complexes. This was consequently associated with reduction in proliferation, IFN-γ production and cytotoxic activity of cytotoxic T lymphocytes (CD8^+^) [98].

A study used the IEDB B-cell epitope prediction tool to detect immune evasion mutations in epitopes along with potential immune cross-reactivity. Several amino acid alignments were found to be similar to human proteins supporting the assumption that molecular mimicry can drive autoimmune cross-reactivity [99].

An epistatic model that predicts mutable sites of SARS-CoV-2 epitopes based on direct coupling analysis (DCA) scores showed that in the RBD of S protein, the predicted mutability was well correlated with experimental measures of protein stability. Four of the positions that displayed high DCA score (indicating high probability for mutation to occur) already are observed in the currently occurring VOCs and VOIs, including positions N501 and E484 [88]. The model proposed nine predictions as highly dangerous positions with high liability to mutate in future SARS-CoV-2 strains, many of them do not occur in current VOCs and VOIs [88].

It is therefore clear that epitope mapping and detection of variants and mutations should continue to provide full understanding of the viral mechanisms, pathogenicity and for providing prediction for SARS-CoV-2 evolutionary effects.

### Evaluation of current vaccines & development of new ones

#### COVID-19 vaccines

Vaccination has been historically known for being the most effective countermeasure for combatting infectious diseases. Hence, one of the top priorities for the containment of COVID-19 pandemic was developing safe and effective vaccines. To date, US FDA approval was granted to at least four types of COVID-19 vaccines. These include: mRNA vaccines, viral vector vaccines, protein subunit vaccines, and inactivated viral particle vaccines. Despite the great effectiveness they showed in slowing down the spread of the virus, they are challenged by the continuous evolution of the virus and the emergence of viral variants sometimes showing higher transmissibility [100–102]. Up to ten-fold reduction in the protective activity of some COVID-19 vaccines against emerging SARS-CoV-2 variants was documented by recent studies [103,104]. This implies an incessant review of COVID-19 vaccines against the emerging viral variants and readiness for implementing the required modifications or developing new ones. A timely approach for developing alternative safe and effective vaccines is the epitope mapping.

#### Epitope-based vaccines

Epitope mapping aids in the selection of epitopes with highest potency in eliciting immune response and could, therefore, be utilized as targets for EBVs production [28]. Mapped epitopes might then be employed in developing vaccines in a process known as “reverse vaccinology” [105]. EBVs are superior to vaccines prepared by conventional methods in several aspects. These include time- and cost- effectiveness and better safety profiles achieved by discarding epitopes with injurious side effects [106,107]. Further, an augmented vaccine potency may be achieved by designing multi-epitope vaccines with reduced risk for pathogens' immune escape [108].

A three-step approach is used in designing EBVs. This starts by prediction of the immunogenic regions, then preparation of the immunogenic construct, and finally evaluating vaccine efficiency [107]. A number of free online tools are available for prediction of B-cell and T-cell epitopes [109]. These provide easier and faster development of EBVs compared with wet lab-based approaches. However, these are often limited by the identification of numerous epitopes that sometimes fail to elicit considerable immune responses in experimental animal models.

#### Role of epitope mapping in developing COVID-19 vaccines

For developing epitope-based SARS-CoV-2 vaccines, several aspects were considered by most of the concerned studies. Even though most of the earlier SARS-CoV-2 vaccine studies focused on triggering humoral immune response [110], concurrent stimulation of B-cell and T-cell immune responses was a common target for most of the recent studies. Stimulation of B-cell immune response for production of nAbs has been known as a major approach for achieving immunity. Neutralizing antibodies against SARS-COV-2 are known to target functional domains of the Spike protein mostly the RBD [67,74]. While the rapid decline in SARS-CoV-2 antibodies was demonstrated in recovered COVID-19 patients by several studies [111,112], up to 2–6 months persistence was reported by others [113–115]. Moreover, the long-term protective effect of the memory B cells was demonstrated after COVID-19 infection [112] and vaccination [116]. Nevertheless, humoral immune response is only part of the immune systems. Cellular immune response plays a dual role in combating viral infections. This comprises the T-cytotoxic cells (CD8^+^)-mediated direct killing of the infected cells as well as the assistance provided by the T-helper cells (CD4^+^) for the B cells to differentiate into plasma cells and produce antibodies [117]. Compared with that of B-cells, the immune response generated by T-cells has been known to be more persistent and less affected by viral mutations [118]. Previous studies have demonstrated less T-cell immune escape by SARS-CoV-2 [95,119–121]. This underscores the need for developing vaccines capable of concomitant induction of B-cell and T-cell immune responses. Only few studies designed candidate vaccines based on T-cell epitopes only [122–124].

Being the most accessible by B-cell receptors, only structural proteins were screened for B-cell epitopes by most groups developing EBVs [47,108,125–127], in particular the Spike protein [47]. Few studies, however, used the whole genome for mining B-cell epitopes [128]. B-cell epitopes were mostly mapped to the S-protein, less frequently they were identified in other structural proteins [125,129].

In the meantime, most researchers scanned the whole genome/proteome (structural and nonstructural proteins) of the virus for detecting T-cell epitopes [125,127,128]. This was rationalized by the fact that both the structural and non-structural proteins are accessible for endogenous processing and presentation by infected cells and thus capable for eliciting cellular immune response [125]. This was also demonstrated in COVID-19 convalescent subjects [130–132]. Yet, structural protein-restricted T-cell epitope prediction was applied by other studies [6,47,108,122,126].

HLA molecules are highly polymorphic among different populations around the world. Hence, the usefulness of EBVs is limited by HLA specificity. It may be impractical to design an EBV that is suitable for large-scale vaccination programs targeting populations with different HLA alleles [133]. In this context, population coverage analysis was part of most SARS-CoV-2 vaccine studies [6,47,110,122,128,134]. This was *in silico* analyzed mostly using the Immune Epitope Database (IEDB) [58] Population Coverage tool. The tool calculates the fraction of individuals predicted to be responsive to the epitopes shortlisted for vaccine development.

Another aspect considered by some authors was variant resistance of candidate vaccines. Some focused on predicting variant-resistant epitopes spanning conserved regions of the virus rather than those most commonly affected by mutations identified in the VOCs [47,135]. Some vaccine development groups were also concerned with the conservancy of the epitopes across different coronavirus species [122,127,134]. An opportunity for combining more than one epitope in one vaccine with different purposes was offered by the multiepitope vaccines. Hence, they had a considerable share of SARS-CoV-2 vaccines development efforts [6,47,110,126,128].

An additional concern in developing COVID-19 epitope-based vaccies is the potential risk of causing exaggerated disease as a result of ADE [136,137]. One approach applied by some research groups to avoid ADE is excludig B-cell epitopes from their proposed vaccines [123].

Avoiding viral epitopes cross reacting with human epitopes is also a matter of concern. Two possible consequences of incorporating such epitopes in candidate vaccines are induction of autoreactive T-cells and/or tolerogenic T-cells that may cause autoimmune response and reduced viral immunogenicity, respectively [138]. This was thoroughly studied by Gustiananda *et al.* during the development of a candidate anti-SARS-CoV-2 epitope-based vaccine. The authors reported a SARS-CoV-2 T-cell epitope “^2784^AIFYLITPV^2792^” cross reacting with an olfactory receptor-derived human peptide “AIFYLITLV” [138]. Mimicry of candidate epitopes to human proteins was also analyzed by other studies as well [139–141].

In addition to the role of epitope mapping in designing EBVs, epitope mapping was also used for evaluation of the protective potential of developed vaccines for eliciting adequate immune response. Yang *et al.* proposed a potential peptide-based vaccine against COVID-19 that could stimulate high and long-lasting antibody response in mice. The authors used the epitope mapping approach to prove the potential of their vaccine for eliciting antibody patterns comparable to those found in COVID-19 convalescent individuals [142]. Epitope mapping was also applied by Conforti *et al.* in preclinical evaluation of a novel DNA vaccine candidate. The vaccine, designated COVID-eVax, is a DNA plasmid that encodes a secreted monomeric form of S protein receptor-binding domain (RBD). The candidate vaccine showed superior immunogenicity to several constructs that express different fractions of the Spike protein designed in the same study. The authors used the ELISpot assay for B cell epitope mapping. In this assay, sera of mice vaccinated by the candidate vaccine were tested against 338 peptides covering the entire S protein that are fixed to ELISA plates. The assay showed that the immunological response in the mice immunized with the candidate vaccine mainly targeted the conserved regions of the RBD rather than those commonly affected by mutations. Hence, the functionality of the candidate vaccine against the currently circulating SARS-CoV-2 VOCs was suggested by the authors. Immunodominant epitopes eliciting T-cell response after immunization by the candidate vaccine were also elucidated [135]. More recently, Liang *et al.* used epitope mapping for evaluation of the spectrum of activity of a lipid nanoparticle-encapsulated mRNA (mRNA-LNP) vaccine that encoded an RBD trimer. Protective humoral and cellular immune responses against SARS-CoV-2 VOCs were generated by the vaccine in infection mouse models. Epitope mapping of T6, a potent mAb generated by the vaccine, was done using Cryo-EM. The mapped epitopes were compared with those deposited in the GISAID database. The analysis revealed a high degree of conservation of the mapped epitope among SARS-CoV-2 variants [143]. Table 2 displays a list of some epitope-based vaccine studies, techniques used, and study outcomes.

**Table 2.** List of some recent epitope-based vaccine studies, techniques used, and study outcomes.

Study	Epitope prediction method	Epitopes identified^†^ (Location^‡^)	Multiepitope vaccine construction	Immunogenicity validation/Epitope mapping of elicited immune response	Ref.
Smith *et al.*	Immuno-informatic tools	6 B (S) 10 T (S, M, and N)	Yes	*In vivo* animal model in mice/ELISA and ELISpot assays.	[106]
Feng *et al.*		19 B (S) 499 T (S, M, and E)			[117]
Qiao *et al.*		7 B (S) 3 T (S)			[138]
Kar *et al.*		15 T (S) 21 B (S)	Yes	*In silico* immune simulation	[125]
Tahir Ul Qamar *et al.*		69 B (M, N, E, ORF6, ORF7a, ORF8, and ORF10) 27 T (M, N, E, ORF6, ORF7, and ORF8)			[133]
Chukwudozie *et al.*		32 B (S) 15 T (S)			[119]
Saba *et al.*		3 B (S) 13 T (ORF1ab, S, M, and E)			[139]
Devi *et al.*		20 B (S, M, N, and E) 79 T (S, M, N, and E)			[127]
Ferreira *et al.*		5 B (S) 6 T (S)			[141]
Adam		1 B (N) 31 T (ORF1a, S, M, and N)			[142]
Rouzbahani *et al.*		14 B (S and N) 14 T (S and N)			[143]
Gustiananda *et al.*		12 T (ORF1ab)	No		[131]
Naz *et al.*		12 T (S) 1 B (S)	Yes	Not tested	[104]
Jyotisha *et al.*		1 B (S) 5 T (S)			[134]
Sanami *et al.*		6 B (S) 12 T (S)			[137]
Fatoba *et al.*		37 T (Orf1ab, S, ORF3a, M, and N) 8 B (Orf1ab, S, ORF3a, M, and N)			[120]
Khan *et al.*		5 B (S, M, N, and E) 21 T (S, M, N, E, ORF1ab, ORF3, ORF6, ORF7, and ORF8)			[121]
Crooke *et al.*		6 B (S, M, and N) 41 T (S, M, ORF1ab, ORF3, ORF6, ORF7, and ORF8)	No		[116]
Alam *et al.*		6 B (S) / 9 T (S)			[132]
Aparicio *et al.*	Experimental Tools	B (S) T (S)	Yes	*In vivo* animal model in mice/ELISA and ELISpot assays	[140]

† B, B-cell epitopes; T, T-cell epitopes.

‡ Viral proteins.

### Epitope-mapping & COVID-19 therapeutics

While vaccination offers the best chance for suppressing the pandemic, a platform for the spread and evolution of the virus is constantly provided by the large pool of unvaccinated individuals. Hence, developing efficient treatment remains one of the forefront priorities for most researchers. It aims at reducing morbidity and mortality due to infection by SARS-CoV-2. Moreover, it may be lifesaving for immunocompromised individuals unresponsive to COVID-19 vaccines [144].

Even though convalescent plasma managed to inhibit SARS-CoV-2 replication and consequently alleviate clinical symptoms of COVID-19 patients [145–147], several practical limitations have been associated with this therapeutic approach. Some of which are the lack of scalability and batch-to-batch variability [148]. Therefore, a good alternative may be provided by monoclonal antibodies (mAbs). These have drawn a considerable attention as promising tools for treatment of numerous diseases, owing to their reliability and high specificity. Therefore, many researchers rushed to develop mAb-based drugs for treatment of COVID-19 [149].

A key step in the characterization of mAbs is the identification of the interacting epitopes [150] using any of the methods described before. For this reason, epitope mapping was an inherent step of almost all studies developing mAbs for therapeutic use. In their effort digging for anti-SARS-CoV-2 mAbs, researchers most commonly screened B-cells of convalescent COVID-19 patients [74,148,151–158], people with past coronavirus infections [159,160], immunized animals [161–164] or phage display libraries [165–167]. A wide variety of epitope mapping techniques were also utilized. Table 3 lists some studies that focused on SARS-CoV-2 epitope-based therapeutics as well as the epitope mapping methods employed in each study.

**Table 3.** List of some recent studies focusing on epitope-based therapeutics and epitope mapping techniques used in each study.

Study	Publication date	Source of mAbs	*In vivo* testing	Epitope mapping method	Ref.
Ju *et al.*	May 2020	Single B cells from individuals infected with SARS-CoV-2	Mouse model	X-ray crystallography	[169]
Brouwer *et al.*	June 2020	Convalescent sera of COVID-19 patients	Not done	Electron microscopy	[155]
Liu *et al.*	July 2020	Convalescent sera of COVID-19 patients	Hamster model	Cryo-electron microscopy	[74]
Tai *et al.*	July 2020	RBD-immunized mice	Not done	ELISA	[163]
Cao *et al.*	July 2020	Convalescent sera of COVID-19 patients	hACE2-transgenic mice	Cryo-Electron Microscopy	[151]
Zost *et al.*	July 2020	Convalescent sera of COVID-19 patients	Mouse model - non-human primate (NHP) model	ELISA	[154]
Baum *et al.*/Hansen *et al.*	August 2020	Convalescent sera of COVID-19 patients and humanized mice immunized by DNA vaccines	Not done	Hydrogen-deuterium exchange mass spectrometry (HDX-MS)	[152,161]
Parray *et al.*	September 2020	Naive human semisynthetic phage library against RBD	Not done	Molecular modeling and docking	[167]
Zhang *et al.*	January 2021	Immunized mice	Mouse model	ELISA	[174]
Kim *et al.*	January 2021	Convalescent sera of COVID-19 patients	Ferret, hamster, and rhesus monkey models	X-ray crystallography	[148]
Piepenbrink *et al.*	March 2021	Convalescent sera of COVID-19 patients	Hamster model	CM-5 sensor chips to which RBD-FC were captured.	[157]
Xie *et al.*	August 2021	Convalescent sera of COVID-19 patients	Not done	ELISA	[153]
Schmitz *et al.*	September 2021	SARS-CoV-2 mRNA vaccine-elicited germinal center B cells	Hamster model	Bio-layer interferometry (BLI)-based competition assays	[205]
Tan *et al.*	November 2021	Naive human phage display antibody libraries	Not done	enzyme-linked immunosorbent assays and surface plasmon resonance analysis	[166]
Yuan *et al.*	December 2021	Large-scale phage libraries for nAbs	Hamster model	Site-directed mutagenesis in RBD followed by screening for mAb binding to cells expressing the mutants.	[165]
Wang *et al.*	December 2021	Convalescent sera of COVID-19 patients	Not done	Cryo-electron microscopy	[175]
Shan *et al.*	December 2021	Convalescent sera of COVID-19 patients	hACE2 Transgenic Mice	Competitive binding of mAbs to SARS-CoV-2 RBD immobilized to a CM5 sensor chip. measured by surface plasmon resonance (SPR)/Cryo-electron microscopy	[158]
Huang *et al.*	January 2022	Convalescent sera of COVID-19 patients	a Syrian hamster model	Cryo-electron microscopy/X-ray crystallography	[184]
Li *et al.*	January 2022	Convalescent sera of COVID-19 patients	Mouse model	Cryo-electron microscopy/cryo-electron tomography/peptide competition/X-ray crystallography	[183]
Kovacech *et al.*	February 2022	Mice immunized with either recombinant S protein or RBD	Mouse model	HDX-MS	[162]
Ueno *et al.*	March 2022	Convalescent sera of COVID-19 patients	Mouse model	ELISA	[156]
Lai *et al.*	March 2022	Mice immunized by purified RBD	Not done	Site-direct mutagenesis in RBD followed by western blot	[164]

The Spike protein of SARS-CoV-2 was found to be a common target for nAbs isolated from COVID-19 convalescent patients [74,151,155,168–170]. Hence, all research efforts were focused on developing mAbs targeting this protein. Epitope mapping analysis of neutralizing mAbs proposed by most studies showed that they interact mostly with RBD [74,148,151–153] and less frequently with non-RBD regions including NTD and S2 domain [74,155,171,172]. Different conformations have been identified for SARS-CoV-2 S protein in which RBD may have an “up” or “down” position. Accordingly, anti-RBD nAbs can be categorized into six major groups briefly discussed by Cui *et al.* class I nAbs bind only to RBDs with “up” position (e.g., LY-CoV016, Brii-196, and CoVOX-150). Class III nAbs bind to RBDs in both “up” and “down” positions (e.g., C121, CoVOX-316, and LY-CoV555). In the patch between the binding sites of class I and III, bind the nAbs that belong to class II (e.g., Regen 10933, COV2-2196, and CoV2-39). The right shoulder of the RBD is targeted by class IV nAbs (e.g., Regen 10987, CoVOX-75, and COV2-2130). When at least one RBD is in the open state, class V (e.g., BD-744, S2H97, and Brii-198) and VI (e.g., DH1047, H014, and S2X35) nAbs interact with two cryptic epitopes common to sarbecoviruses [173]. The potential of the anti-RBD mAbs for species-specific inhibition was anticipated by Ju *et al.* [169]. This was supported by demonstrating cross reactivity between SARS-CoV-2 mAbs with the trimeric Spike proteins of SARS-CoV and MERS-CoV but not with their RBDs. The authors then suggested that cross reactivity was mediated by epitopes located outside the RBD regions [169]. In contrast, cross reaction between anti-SARS-CoV RBD mAbs and SARS-CoV-2 RBD was reported by Zhang *et al.* [163]. Two anti-SARS-CoV RBD mAbs cross reacted with SARS-CoV-2 RBD protein and could cross-neutralize infection by SARS-CoV-2 S pseudovirus. ELISA-based Epitope-mapping revealed that they interacted with epitopes that were conserved between the two species. Other studies also reported cross reaction between mAbs against RBD of SARS-CoV-2 and SARS-CoV [174,175]. In addition, mAbs that cross react with human coronaviruses have been recently isolated [176,177]. These target the conserved S2 domain of coronaviruses.

Emergency use authorization has been granted to several mAbs targeting RBD of the Spike protein while others are still in the pipeline [178]. Nevertheless, these are facing a great challenge by the emergence of VOCs carrying Spike protein mutations. A lot of studies have reported the resistance of some variants to the currently available therapeutic antibodies [179–182]. To this end, one target of several recent studies was to develop mAb showing activity against the currently circulating VOCs [148,162,183,184]. A database was recently created for housing published data on the susceptibility of SARS-CoV-2 variants to neutralization by mAbs and plasma obtained from convalescent patients and vaccinated persons. In addition, Spike mutations selected *in vitro* or *in vivo* in the presence of mAbs are recorded [185].

As for other RNA viruses, SARS-CoV-2 is expected to exhibit a high mutation rate under the selective pressure of therapeutic mAbs. A countermeasure for the mutational escape of the virus is developing mAb cocktails [144,152,162,186]. Of them, much interest was earned by those targeting non-overlapping epitopes for which simultaneous mutations at two distinct genetic sites are required for immune escape [152,174]. Interestingly, mutational escape of SARS-CoV-2 in presence of individual mAbs was found to be inhibited by cocktails of the same mAbs [152]. Several candidate anti-SARS-CoV-2 mAb cocktails were, thus, proposed for treatment of COVID-19 [152,162,174]. Apart from the immune escape, anti-SARS-CoV-2 mAb cocktails were also reported to achieve virus neutralization synergism. This allows the use of lower doses of individual mAbs to achieve the same neutralization potency of each [154]. Selection of mAbs with non-overlappig epitopes for incorporation in cocktails is mostly based on epitope competition or binning assays [152,162,174]. In this type of assays, candidate mAbs are tested in a pairwise manner for simultaneous binding to a specific antigen. While not defining the exact location of the epitopes targeted by a mAb, a blocking profile for each mAb can be generated by epitope binning assays. This shows the possible interference between mAbs before use in cocktails [187,188].

Recombined antibodies harboring heavy and light chains from different nAbs also showed promising results. Xie *et al.* reported significantly higher neutralizing ability for the recombined antibodies against mutant pseudoviruses than mAbs from which they were originally derived [153].

Similar to vaccines, ADE risk is also associated with the admnistration of mAb as therapeutic agents for COVID-19 [136,137]. This was considered by some studies during selection of candidate therapeutic mAbs [174,189].

### Epitope mapping & SARS-CoV-2 diagnostics

Although nucleic acid amplification tests (NATs) are currently known as the gold standard for COVID-19 diagnosis, rapid, simple, reliable, and low-cost point-of-care diagnostic tests (POCTs) are still required. These may facilitate immediate and on-site diagnostic decisions as well as mass-screening of potential SARS-CoV-2 infected cases [190,191]. One of the convenient methods is viral antigen detection tests. These allow direct demonstration of SARS-COV-2 antigens in clinical specimens of infected individuals in less than 30 minutes. Owing to their high specificity and reliability, monoclonal antibodies (mAbs) precisely targeting specific viral antigens are often utilized in antigen detection tests. One feature affecting the performance of an antigen detection test is employing high-quality monoclonal antibodies precisely targeting specific viral antigens [192]. This is consistently determined through epitope mapping that allows accurate identification of key amino acid residues involved in antibody recognition and binding [150].

A good candidate for SARS-CoV-2 mAb-based antigen detection kit is the nucleocapsid protein (N). It is a predominant structural protein exhibiting several promising competencies for immune-based diagnostic tools. It is strongly immunogenic and highly conserved [193]. It is also detectable in various clinical samples of COVID-19 patients that include gargle solution, nasopharyngeal aspirate, serum, fecal material as well as urine [194]. Even though N protein is located within the viral particle, it was found to elicit higher and earlier antibody response in COVID-19 patients than do the Spike protein [195]. A major drawback of the N protein, however, is the high homology to N proteins of other human coronaviruses. Cross reactivity is hence expected, negatively affecting the specificity of the antigen detection tests incorporating mAb targeting SARS-CoV-2 N protein [192].

To this end, several studies have focused on using epitope mapping for producing mAbs that exclusively react with SARS-CoV-2 with no cross-reactivity with other human coronaviruses. Yamaoka *et al.* produced a nucleocapsid protein devoid of the amino acid residues showing sequence homology to other human coronaviruses. The protein was used as an immunogen to produce SARS-CoV-2-sepcific mAbs. The epitopes revealed by the ELISA-based epitope mapping were used to confirm the antigenic discrimination potential of the candidate mAbs. This was achieved by multiple sequence alignments of N proteins derived from various human coronaviruses. The candidate mAbs were found to exclusively react with the N proteins of SARS-CoV-2 including the three major VOCs [192].

Less stringency was applied by Tian st al. in developing mAb candidates for antigen detection diagnostic kits. They generated a rabbit mAb (RAb) against SARS-CoV-2 N protein that cross-reacts with the N protein of SARS-CoV but not that of common human coronaviruses including MERS-CoV. The specificity of RAb was confirmed by epitope mapping against truncated N protein recombinant proteins using indirect-ELISA and western blot assays [196]. Using insect cell-based expression systems and recombinant N protein as immunogen, six anti–N protein mAbs were prepared by Tian *et al.* These showed high titers and good affinity for N protein of SARS-CoV-2. Further, epitope-mapping was done against overlapped peptides covering SARS-CoV-2 N protein using peptide ELISA and dot-blot assays. The analysis revealed a novel linear B -cell epitope that showed high conservancy among SARS-COV-2 strains from different geographic regions as well as VOCs and VOIs strains [197].

An epitope mapping platform was created by Liang T, Cheng M, Teng F*et al.* [72] using a peptide-based proteome microarray containing hundreds of peptides representative of all proteins of SARS-CoV-2. Their analysis included epitope mapping of commercial antibodies to structural and nonstructural proteins. The epitope landscape of SARS-CoV-2 antibodies was thereby characterized providing a resource for developing reagents for COVID-19 diagnosis and treatment.

Epitope prediction immunoinformatic tools were recently utilized for developing a mAb-based biosensor for SARS-CoV-2. The biosensor incorporated a pair of mAbs that bind to conserved epitopes on SARS-CoV-2 nucleoprotein. The sensitivity and specificity of the biosensor were tested using recombinant N protein and clinical samples from real time PCR-confirmed COVID-19 cases [198].

## Conclusion

With numerous available methods, epitope mapping has been widely applied in the scientific research for combating COVID-19. Epitope mapping have been utilized for SARS-CoV-2 variant analysis, immune-profiling, mutability monitoring, and developing immune-based vaccines, therapeutics and diagnostics. Some studies utilized wet lab-based epitope mapping techniques, but others were confined to computer-based in silico analyses. While in silico analysis have been known to save time and cost for getting timely solutions for this deadly pandemic, results should be further confirmed by wet lab-based experiments.

## Future perspective

The pivotal role of epitopes in eliciting immune response makes epitope identification essential to increase the understanding of the mechanisms underlying antigen recognition by antibodies, hence improving the design of therapeutic or diagnostic mAbs and epitope-based vaccine [29]. Continuous investigation of epitopes and their possible roles thus represent the most important aspect in future studies. *In silico* prediction-based methods represent an alternative to experimental methods allowing - in many times - precise determination of epitopes along with prediction of possible mutations and their effects on immune response [42]. Validation of the *in silico*-predicted epitopes, however, remains a necessary step.

Approval of COVID-19 EBV represents another main aspect in future studies. While several EBV candidates were developed for COVID-19, none have yet been approved by the U.S FDA. Further studies are also still required to provide understanding for the long COVID-19 phenomenon and the possible epitopes related to it.

**Executive summary**

- Epitope is the part of the antigen which can generate immune response following its recognition by either the host B- or T-cells or related molecules (antibodies, MHCs, etc.).
- The pivotal role of epitopes in eliciting immune response makes epitope identification essential to increase the understanding of the mechanisms underlying antigen recognition by antibodies, hence improving the design of therapeutic or diagnostic mAbs and epitope-based vaccine.

**Methods of Epitope mapping**

- The amino acid residues constituting peptide epitope can present either in continuity (linear or sequential) or as a surface patch (conformational or discontinuous).
- Linear epitopes depend on the amino acid sequence in a random coil form of a peptide from the protein while conformational ones depend on the sequence of residues brought together by folding of protein molecules.
- Epitope mapping provides information about the corresponding immune response. Several methods have evolved which allow precise determination of specific peptide sequences responsible for eliciting B-cell and T-cell activity in the affected patients.
- While B-cell epitope prediction seems a straightforward process, the high degree of MHC polymorphism makes T-cell epitope prediction challenging.
- *In silico* prediction-based methods represent an alternative to experimental methods allowing - in many times - precise determination of epitopes along with prediction of possible mutations and their effects on immune response. Validation of the *in silico*-predicted epitopes, however, remains a necessary step.
- Extensive amount of data has urged the presence of databases for epitope mapping. Among which, the highest popularity was reserved to the Immune Epitope Database (IEDB).

**Applications of epitope mapping in COVID-19**

- Studies relating epitope-antibody response to disease outcomes showed interesting findings justifying the broad spectrum of severity observed in COVID-19 patients.
- One prominent study on this subject was the one that reported an inverse correlation between the total predicted MHC-I epitope and COVID-19 mortality.
- In addition, epitope mapping made possible monitoring of the evolution of the virus and its impact on the effectiveness of the established immune-based countermeasures.
- EBVs provide more effective and safe alternatives to the conventional vaccines due to harboring the least number of the optimally immunogenic epitopes. While several EBV candidates were developed for COVID-19, none have yet been approved by the U.S FDA.
- Most of the candidate EBVs comprised both B- and T-cell epitopes for concomitant induction of humoral and cellular immune responses.
- Epitope-based COVID-19 therapeutics were also optimized through epitope mapping for broad spectrum effectiveness against human coronaviruses and SARS-COV-2 VOCs; suppressing viral mutational immune escape; and lack of ADE potential.
- Selection of SARS-CoV-2 mapped epitopes for incorporation in diagnostic kits targeted epitopes fulfilling certain criteria. These include: early detectability, conservation among SARS-CoV-2 strains including VOCs, abundance in clinical samples, and finally species-specificity.
